# Three-Factor Kinetic Equation of Catalyst Deactivation

**DOI:** 10.3390/e23070818

**Published:** 2021-06-27

**Authors:** Zoë Gromotka, Gregory Yablonsky, Nickolay Ostrovskii, Denis Constales

**Affiliations:** 1 Department of Electronics and Information Systems, Ghent University, 9000 Ghent, Belgium; denis.constales@ugent.be; 2 Department of Energy, Environmental & Chemical Engineering, McKelvey School of Engineering, Washington University, St. Louis, MO 63130, USA; gregoryyablonsky@gmail.com; 3 Euro Gas, 24000 Subotica, Serbia; n-ostr@yandex.ru

**Keywords:** catalyst deactivation, kinetic equation, reversible deactivation and aging, separability

## Abstract

The three-factor kinetic equation of catalyst deactivation was obtained in terms of apparent kinetic parameters. The three factors correspond to the main cycle with a linear, detailed mechanism regarding the catalytic intermediates, a cycle of reversible deactivation, and a stage of irreversible deactivation (aging), respectively. The rate of the main cycle is obtained for the fresh catalyst under a quasi-steady-state assumption. The phenomena of reversible and irreversible deactivation are presented as special separate factors (hierarchical separation). In this case, the reversible deactivation factor is a function of the kinetic apparent parameters of the reversible deactivation and of those of the main cycle. The irreversible deactivation factor is a function of the apparent kinetic parameters of the main cycle, of the reversible deactivation, and of the irreversible deactivation. The conditions of such separability are found. The obtained equation is applied successfully to describe the literature data on the reversible catalyst deactivation processes in the dehydration of acetaldehyde over $TiO_{2}$ anatase and in crotonaldehyde hydrogenation on supported metal catalysts.

## 1. Catalyst Deactivation: Categories and Factors

Catalyst deactivation is a complex, non-steady-state process governed by a variety of phenomena influenced by many physicochemical factors. Different categories of catalyst deactivation have been introduced, such as chemical poisoning, fouling (e.g., coke generation), thermal deactivation, and mechanical degradation [1]. From another perspective, different primary categories of catalyst deactivation can be proposed:

1. Reversible and irreversible deactivation;
2. Chemical and physical deactivation;
3. ‘Intrinsic’ and ‘extrinsic’ deactivation.

Chemical deactivation is defined as the process caused by a set of chemical transformations. Physical deactivation is a result of one or several structural and mechanical changes, e.g., sintering, and surface and bulk phase transitions, which are responsible for a change in the number of active sites.

‘Intrinsic’ deactivation can be defined as a process caused by reactants and products of the main reaction within a chosen domain of working parameters, i.e., chemical composition, temperature, and pressure. Consequently, ‘intrinsic’ factors of deactivation include the concentrations of reactants and products of the main reaction and the temperature. Conditions of preliminary catalyst preparation and pretreatment can be considered ‘intrinsic’ factors of a catalyst as well.

‘Extrinsic’ deactivation is a consequence of the influence from factors beyond the main process and its conditions, e.g., poisons and impurities, excessive temperature, pressure, and flow-rates.

In more detailed categorizations, these factors can be combined and coupled. Further, additional processes of catalyst activity evolution can be considered, e.g., catalyst self-regeneration as a result of interaction between the deactivated catalyst with some ‘intrinsic’ reactants/products; catalyst ‘forced’ deactivation, such as reoxidation during the regeneration period. More rigorously, catalyst activation before the starting regime can be included in the types of processes that determine catalyst activity.

## 2. Phenomenological and Semiphenomenological Models of Catalyst Deactivation: State-of-the-Art

Within the phenomenological approach, the main characteristic of the catalytic process is the catalytic reaction rate (*r*), which depends on the concentrations of reactants ($C=C_{1},C_{2},\ldots$), temperature (*T*), and catalyst activity (*a*),

(1) $$r=f_{r}\left(C,T,a\right).$$

The catalyst activity *a* is considered a function of the reaction conditions, here *C* and *T*, and its change can be termed as ‘catalyst deactivation’.

Szepe and Levenspiel were the first to use such a phenomenological approach [1]. They proposed the following phenomenological deactivation equation:

(2) $$\begin{array}{cc} r(t)= & r^{0}a\left(t\right), \end{array}$$

(3) $$\begin{array}{cc} \frac{da}{dt}= & -f\left(C,T\right)a^{d}, \end{array}$$

where $r^{0}$ is the reaction rate over the non-deactivated (‘fresh’) catalyst and *d* is an empirical parameter.

The main assumption of this model is that the reaction and deactivation kinetics are *separable*. The function f(C,T) can be empirical, or reflect the rate of deactivation according to its assumed power-law decay kinetics.

Then, Corella et al. [2,3] analyzed the empirical parameters relevant to Equation (1), resulting in the following expression:

(4) $$\frac{da}{dt}=-k_{d}C_{i}^{n}a^{d},\;d=\frac{m+h-1}{m},$$

where *m* and *h* are the number of active sites involved in the limiting steps of the reaction and of the deactivation, respectively, while *n* is an empirical parameter.

In [4], the authors presented the kinetic model with a description of two different periods of irreversible deactivation.

## 3. Modified Phenomenological Models of Catalyst Deactivation

In catalytic literature, many models combining phenomenology and some mechanistic considerations of deactivation have been presented, e.g., power-law kinetic dependencies and Langmuir–Hinshelwood relationships based on the concept of adsorption equilibria, see Butt [5] and Bartholomew [6]. Such models can be termed as *semiphenomenological*.

In 1989, Ostrovskii and Yablonskii proposed the semiphenomenological model of single-route catalytic reactions assuming two types of catalyst deactivation, i.e., reversible and irreversible (‘aging’) [7]. In deriving this model, the known principle of quasi-steady-state (QSS) concentrations was used to obtain the concentration of the catalytic intermediate, which deactivates during the process.

In classical chemical kinetics, this principle regarding the intermediates of a complex chemical reaction is very popular. It is attributed to Bodenstein [8] and sometimes Chapmen as well [9]. The physicochemical foundation of the QSS principle is a separation in time scales, which is caused by the hierarchy in the parameters of kinetic models.

There are two types of such hierarchy:

1. A large difference between different kinetic coefficients;
2. A large difference between the total amount of main reactants and the total amount of intermediates.

Otherwise, for a ‘gas–solid’ catalytic reaction the total number of active catalytic centers is much smaller than the total number of gas molecules (see Chapter 3, [9]).

The second hierarchy is specific for heterogeneous catalytic reactions [9]. In the pioneering paper by Michaelis and Menten, both hierarchies were considered [10]. Recently, results of this paper were revisited and generalized by Gorban [11]. The general mathematical theory of different asymptotic regimes in chemical kinetics was presented by Gorban et al. as ‘Asymptotology’ [12].

**Figure 1:**
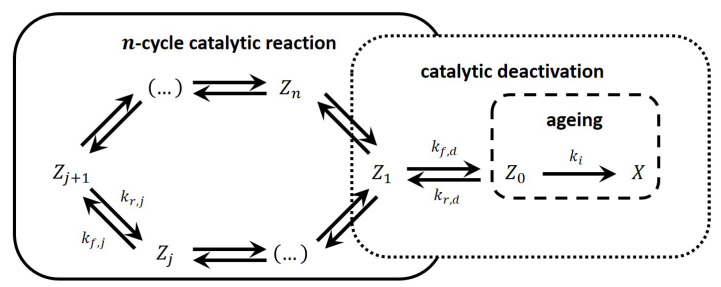
The three-building-block scheme approach to phenomenological modeling for a linear catalytic reaction accompanied by linear catalyst deactivation. Block one is a *n*-step linear catalytic reaction. Block two is a linear reversible catalyst deactivation. Block three is aging, i.e., linear irreversible catalyst deactivation.

Mathematically, the hierarchy between parameters of a model creates so-called “small parameters” within the subsystem of differential equations belonging to the catalytic intermediates. Then, this subsystem transforms into a subsystem of algebraic equations. Consequently, concentrations of the intermediates are presented as functions of model parameters. For heterogeneous catalytic reactions, concentrations of intermediates are very small. Basically, it is the distinguishing feature of catalytic intermediates in the QSS regime. Later, Ostrovskii developed this approach further in the monograph [13] and the paper [14]. We will present this approach in detail, since the goal of our paper is to modify and generalize it. In all cases analyzed in [7,13,14], a detailed linear mechanism was considered, i.e., only one ‘molecule’ of the catalytic intermediate participants in all reactions of a n-step single-route mechanism. In [7,9,14], the catalytic reaction accompanied by reversible deactivation and aging is presented by the three-building-block scheme (Symbols adapted to match current article conventions.) in Figure 1.

For the presented scheme, the following hierarchy of rates is maintained:

(5) $$r\gg r_{d}\approx r_{s}\gg r_{a},$$

where *r*, $r_{d}$, $r_{s}$, and $r_{a}$ are the rates of the reaction, deactivation, self-regeneration, and aging, respectively; $r^{0}$ is an observed reaction rate over the non-deactivated (‘fresh’) catalyst.

(6) $$\begin{array}{cc} r=f_{r}\left(C,T,Z_{i}\right), & \;\;\;\;1\leq i\leq n, \end{array}$$

(7) $$\begin{array}{c} r_{d}=f_{d}\left(C,T,Z_{1}\right), \end{array}$$

(8) $$\begin{array}{cc} r_{s}= & f_{s}\left(C,T,Z_{0}\right), \end{array}$$

where $Z_{1}$ is the dimensionless concentration of the first intermediate of the catalytic cycle, the intermediate of which in this scheme is the point of deactivation; $Z_{0}$ and *X* are dimensionless concentrations of catalytic intermediates, which are excluded from the reaction cycle (deactivated and aged part of the catalyst, respectively); $k_{f,i}$, $k_{f,d}$, $k_{r,d}$, and $k_{irr}$ are the apparent kinetic parameters of the corresponding steps ($k_{f,i}=r_{f,i}/Z_{i}$), so that $k_{f,i}$, $k_{f,d}$, $k_{r,d}$, and $k_{i}$ are functions of the temperature and concentrations according to the mechanism.

The unit of these rates here and further in this paper is $s^{-1}$. The traditional unit for rates, ${\mathrm{mol}}_{\mathrm{gas}}.cm_{\mathrm{cat}}^{-3}s^{-1}$ can be easily converted into $s^{-1}$.

The unit for rates, ${\mathrm{mol}}_{\mathrm{gas}}.cm_{\mathrm{cat}}^{-2}s^{-1}$, can be converted into $s^{-1}$ as well, based on the known catalyst surface per gas volume.

Apparent kinetic coefficients can be treated as the rates of corresponding reactions at the unitary concentrations of corresponding intermediates. They may include concentrations of gas species as factors. Apparent kinetic parameters depend on the temperature in accordance with the Arrhenius law.

For the analysis of concrete cases, this equation will be presented as a function of concentrations and temperature. We consider using such a form in further studies, see Appendix A.

Regarding the characteristics of kinetic model (6)–(8), two groups of them can be distinguished:

1. Characteristics that are measured experimentally (rate, *R*; temperature, *T*; concentrations, $C_{i}$; relative catalyst activity, *a*);
2. Characteristics that are calculated (concentrations of intermediates, $Z_{j}$).

Intermediates of the main reaction are obtained as functions of measured characteristics using the principle of quasi-steady-state concentrations. Then, intermediates related to catalyst deactivation are calculated via the corresponding model of differential equations. Then, the kinetic model is supplemented by the equation of mass balance of the laboratory reactor.

Usually, the rate of reversible deactivation is $10^{-2}$∼$10^{-4}$ times the rate of the catalytic cycle. Similarly, the rate of catalyst aging is much slower than the rate of reversible deactivation (5). Therefore, it is considered that the reaction cycle is in a QSS regime with respect to the reversible deactivation process. Further, the deactivation process can be treated as a QSS one with respect to the aging process.

The temporal change of the catalyst’s deactivated form is presented as follows:

(9) $$\frac{dZ_{0}}{dt}=r_{f,d}-r_{r,d}=k_{f,d}Z_{j}-k_{r,d}Z_{0}.$$

Due to the QSS regime of the reaction,

(10) $$\begin{array}{cc} \sum_{i=1}^{n}Z_{i}= & 1-Z_{0}, \end{array}$$

(11) $$\begin{array}{ccc} Z_{i}\left(t\right)= & Z_{i}^{0}\left(1-Z_{0}\left(t\right)\right), & \;\;\;\;i=1,\cdots ,n, \end{array}$$

where $Z_{i}^{0}$ is the coverage of the *i*-th intermediate over the non-deactivated (‘fresh’) catalyst. Note that *a* is defined as the relative catalyst activity, see Equation (2),

(12) $$\begin{array}{cc} a= & r/r^{0}, \end{array}$$

(13) $$\begin{array}{cc} a(t)= & 1-Z_{0}\left(t\right), \end{array}$$

thus, Equation (9) can be transformed into

(14) $$-\frac{da}{dt}=\frac{r^{0}}{k_{f,j}}k_{f,d}a-k_{r,d}\left(1-a\right).$$

In [13,14], Equation (14)—regarding solely reversible deactivation—is termed as a *general deactivation equation for linear catalytic mechanisms*. At the final state, $\frac{da}{dt}=0$,

(15) $$\begin{array}{cc} \frac{r^{0}}{k_{f,j}}k_{r,d}a_{s}= & k_{r,d}\left(1-a_{s}\right), \end{array}$$

(16) $$\begin{array}{cc} k_{r,d}= & \frac{r^{0}}{k_{f,j}}k_{f,d}\frac{a_{s}}{1-a_{s}}. \end{array}$$

Parameter $a_{s}$ corresponds to the steady-state of the reversible deactivation process, i.e., the so-called residual activity that is achieved when the rate of deactivation ($r_{f,d}=k_{f,d}Z_{j}$) and the rate of self-regeneration ($r_{r,d}=k_{r,d}Z_{0}$) are equal.

**Figure 2:**
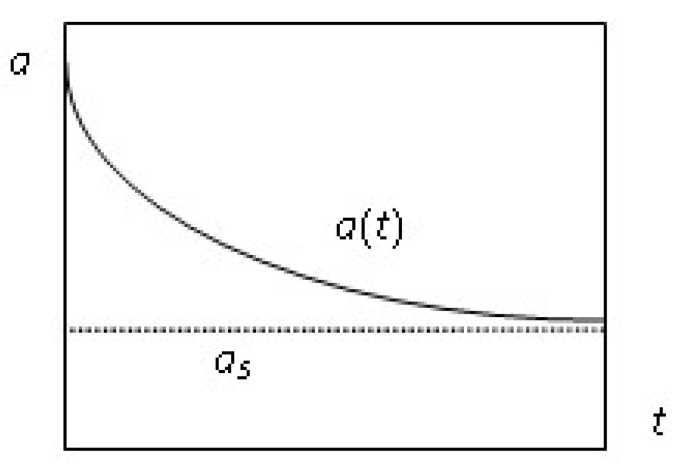
Illustration of residual activity $a_{s}$.

Practically, in accordance with the statement presented in [13,14], this happens at some $t=t_{s}$, when $a\left(t_{S}\right)=a_{s}\pm \varepsilon$ with the accuracy of the experiment (Figure 2).

Substituting $k_{r,d}$ from Equation (16) into Equation (14), another form of catalyst deactivation equation is obtained

(17) $$\begin{array}{cc} r(t)= & r^{0}a\left(t\right), \end{array}$$

(18) $$\begin{array}{ccc} \frac{da}{dt}= & -\frac{r^{0}}{k_{f,j}}k_{f,d}\frac{a-a_{s}}{1-a_{s}}, & t<t_{s}. \end{array}$$

It is stated that this form is more convenient for the interpretation of experimental data on reversible deactivation [7,13,14].

Then, for the aging process ($t>t_{s}$), using the separate equation was proposed [13,14],

(19) $$\frac{da}{dt}=-\frac{r^{0}}{k_{f,j}}k_{i}\frac{a_{s}}{1-a_{s}}a,\;t>t_{s}.$$

This is valid in the period after achieving the level of residual activity $a_{S}$. The solution of Equation (18) is a simple exponential dependence:

(20) $$\begin{array}{cc} r(t)= & r^{0}a\left(t\right), \end{array}$$

(21) $$\begin{array}{ccc} a\left(t\right)-a_{s}= & \left(1-a_{s}\right)\exp\left(-\lambda t\right), & \;\;\;\;\lambda =\frac{r^{0}k_{f,d}}{k_{f,j}\left(1-a_{s}\right)}. \end{array}$$

## 4. Goals of the Paper

One goal of this paper is to develop the theory of catalytic kinetics accompanied by reversible and irreversible deactivation. We will rigorously answer the question of whether it is possible to present the kinetic equation in which the factors of main cycle, reversible, and irreversible deactivation (aging) are separated. The result of our analysis will be obtained for a main cycle that is a single-route mechanism, linear regarding the catalytic intermediates, and under quasi-steady-state assumptions. The reversible deactivation cycle will be taken as a one-step reversible mechanism, and the irreversible deactivation as a single irreversible step. The form and the conditions of separability of the deactivation factors will be analyzed.

## 5. Catalyst Deactivation as a Complex Process: A Graphical Example

In the phenomenological model, the kinetic model is split into the main reaction kinetics and the deactivation kinetics. It makes use of a *separability* assumption on these two kinetic components. In this section, the simplified catalyst deactivation scheme will be presented to highlight the features of a *separable* model. Such model types are used to describe the different processes with catalyst deactivation, e.g., [15,16].

The three-building-block scheme in Figure 1 shows along which lines the phenomenological model tries to implement *separability* assumptions. These are obviously based on the QSS assumptions of the catalytic cycle mentioned above. While the depicted scheme is completely linear with respect to the catalyst intermediates, the kinetic parameters in this scheme are apparent, i.e., they may include concentrations of reactants or products. For simplicity reasons, the kinetic parameters are assumed to be constant, so that the model may be treated completely linearly.

In the section, we look at a specific 2-step example. The elementary reaction steps below describe the catalyst behavior of this example:

1. $Z_{1}+A\overset{k_{f,1}}{\underset{k_{r,1}}{⇄}}Z_{2}$;
2. $Z_{2}\overset{k_{f,2}}{\underset{k_{r,2}}{⇄}}Z_{1}+P$;
3. $Z_{1}\overset{k_{f,d}}{\underset{k_{r,d}}{⇄}}Z_{0}$;
4. $Z_{0}\overset{k_{i}}{\to }X$.

In this model, chemical A is the reactant and chemical P is the product. The inflow and outflow of these two chemicals are assumed to keep the concentration of each constant. This extra assumption has the added benefit of turning this model completely linear. For this reason, we introduce the new parameters $k_{f,1}=\left[A\right]\kappa_{f,1}$ and $k_{r,2}=\left[P\right]\kappa_{r,2}$ and, with this, we can indeed confirm that this reaction scheme is equivalent to Figure 1 with n=2.

For this example and the remainder of this article, the catalyst intermediate $Z_{1}$ is the point of deactivation and holds an initial relative concentration of one, while all other intermediates initially have a concentration of zero.

**Figure 3:**
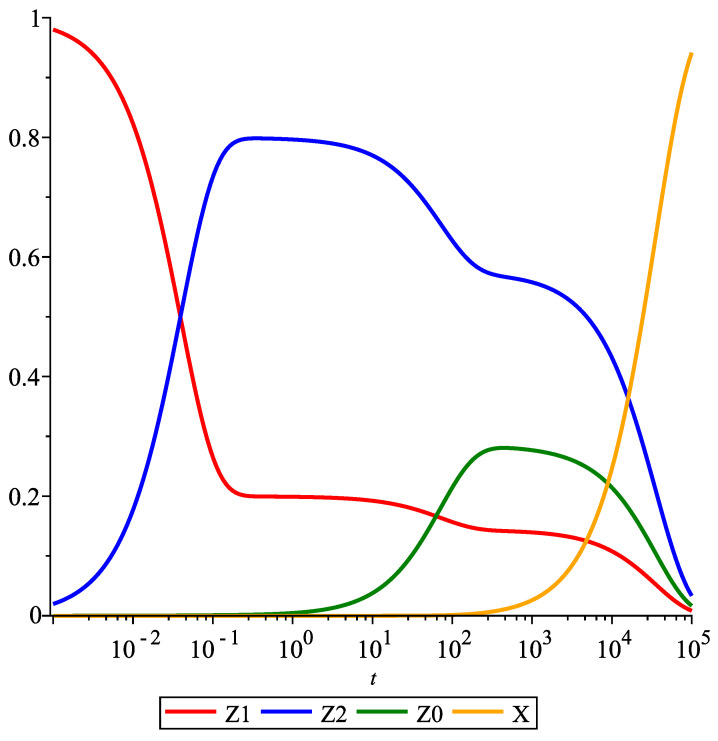
Concentration profiles of a 2-step catalytic reaction with linear deactivation. Parameter values are taken as follows: $k_{1}=20$, $k_{2}=5$, $k_{3}=0.02$, $k_{4}=0.01$, and $k_{5}=0.0001$.

Appendix B notes the mathematically exact solution for the rate equation given these assumptions and conditions on the model. Below, Figure 3 shows the graphics of intermediates’ dynamics, with linear kinetics both in the main reaction as well as in the deactivation. The catalyst intermediates Zi (i=0,1,2) seem to have several plateaus within the course of the reaction. Intermediates $Z_{1}$ and $Z_{2}$ belong to the main reaction. They appear to have two plateaus before they fully reduce to zero.

**Figure 4:**
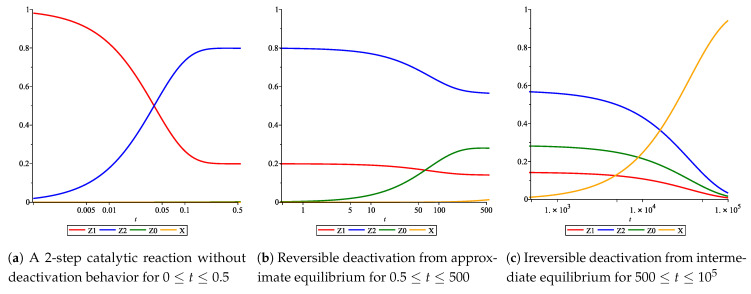
Concentration profiles of a 2-step catalytic reaction with linear deactivation, split by separability. Parameter values are taken as follows: $k_{1}=20,\;k_{2}=5,\;k_{3}=0.02,\;k_{4}=0.01,\;k_{5}=0.0001$.

The kinetic behavior up to the first plateau, see Figure 4a, can be argued to approximate the behavior of a catalyst without deactivation. This claim is explored in Section 6. Furthermore, the graphs in Figure 3 and Figure 4 have a log-scale on their time-axes. As such, the time in which the kinetic behavior mimics that of the catalyst without deactivation is relatively negligible, specifically, when one is interested in the deactivation behavior of the catalyst.

If the graph in Figure 4a resembles that of a catalyst without deactivation, then, by extension, the remaining graphs embody the deactivation. As discussed previously, deactivation may be categorized by reversibility. A reversible deactivation would shift one plateau value to another, while an irreversible deactivation would shift a plateau to zero. By these attributes, we may conclude that Figure 4b shows a reversible deactivation of the catalyst and Figure 4c shows an irreversible deactivation, i.e., aging, of the catalyst. In Section 7, we show that under the right conditions, reversible and irreversible deactivation can also be represented by a *separable* model.

The log-scale is necessary to observe all three segments within one graph. Segment one (Figure 4a) happens quickly and the exact curve can be negligible when studying deactivation. Segment two (Figure 4b) happens less-fast and segment three (Figure 4c) is the slowest. Clear separation of these segments graphically—i.e., in time—is what extends into the *separability* of the mathematical model.

## 6. Modeling of the Main Catalytic Cycle

When modeling a catalytic reaction, one must account for the underlying reaction mechanism. Elementary reaction steps need to be identified and assumptions are made about the catalytic intermediates, one of which is the quasi-steady-state (QSS) assumption. The catalytic cycle is assumed to stabilize quickly and models will describe the rate in terms of reactants and products.

However, these types of approaches usually do not include the possibility for catalyst deactivation. Catalyst properties like activity and selectivity are assumed constant under the right conditions, even though the deactivation of a catalyst results in the decline of these properties (usually) over the long term.

This section will discuss the integration of deactivation with the standard model. It further explores the compatibility of the QSS assumption with the modeling of slow catalyst deactivation.

### 6.1. Properties and Assumptions of the Main Catalytic Cycle

The (catalytic) reaction mechanism is the collection of elementary steps that describe the process of going from reactant A to product P, i.e., the overall reaction

A→P.

Such an overall reaction does not include catalytic intermediates. The main catalytic cycle is characterized by this overall reaction and is a collection of elementary steps that involve catalyst intermediates $Z_{i}$, i=1⋯n. In this report, the collection of elementary steps is assumed to be cyclic. There can be catalyst intermediates and, by extension, elementary steps that do not contribute to this overall reaction; as such, these intermediates are considered to be outside the main catalytic cycle and are hence neglected.

**Figure 5:**
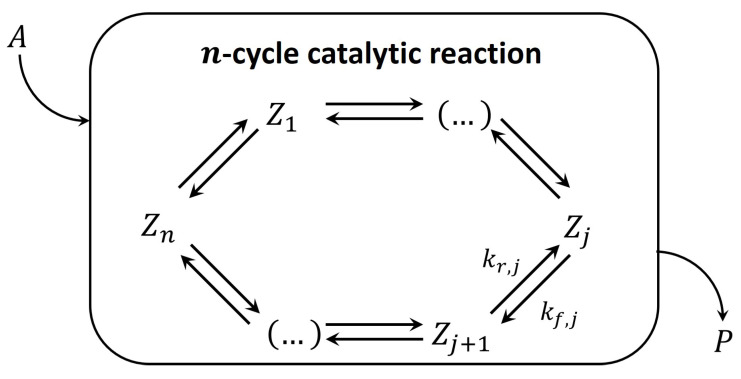
An example scheme for a main catalytic cycle in the linear *n*-step form.

The main cycle in Figure 5 consists of a set of elementary reactions in which catalytic intermediates are participating. This set is called a detailed mechanism of cyclic catalytic reactions. Here, the elementary steps include only one molecule of the intermediate, and mechanisms with this property are termed ‘linear’. However, note that in this paper, the kinetic parameters are apparent, i.e., they may include concentrations of reactants or products as factors. For the rest of this paper, Figure 5 is assumed to be the standard form of the main cycle. Any model based on this main cycle will not provide the whole picture, i.e., it disregards possible deactivation. However, if the deactivation is comparatively slow, the main catalytic cycle is a good model for the kinetic properties in the short term. Since slow deactivation is not observable in the short term, it can be assumed negligible on this time scale. As such, we have separated the main catalyst reaction from catalyst deactivation. Fast deactivation most often affects the kinetics of the main catalytic cycle in the short term and, thus, cannot be ignored. For this article, the focus is on slow deactivation to ensure *separability*.

Similar to the previous papers [7,13,14], here, the quasi-steady-state (QSS) principle is used in combination with the *separability* assumption. This principle results in the corresponding algebraic equation

(22) $${\dot{Z}}_{j}=\sum_{i}{\left(r_{i}\right)}_{f}-\sum_{i}{\left(r_{i}\right)}_{r}\approx 0,$$

for any given intermediate $Z_{j}$.

With this information, we have the tools to detect and analyze any main *n*-step reaction. Rates and concentrations can be determined up to a reasonable accuracy.

### 6.2. Determining the Rate of the Main Catalytic Cycle

While most of the analyses performed in this article can be extended to any catalytic mechanism with linear deactivation, here, we keep to a scheme that is linear in both the main cycle as well as the deactivation. Taking another look at the example from Section 5 we will discuss these different points from a mathematical approach:

1. $Z_{1}\overset{k_{f,1}}{\underset{k_{r,1}}{⇄}}Z_{2}$;
2. $Z_{2}\overset{k_{f,2}}{\underset{k_{r,2}}{⇄}}Z_{1}$;
3. $Z_{1}\overset{k_{f,d}}{\underset{k_{r,d}}{⇄}}Z_{0}$;
4. $Z_{0}\overset{k_{i}}{\to }X$.

In this model, the reactant *A* and the product *P* are part of the apparent kinetic parameters $k_{f,1}$ and $k_{r,2}$, respectively.

Here, steps 1 and 2 are the main catalytic cycle, step 3 is (slow) reversible deactivation, and step 4 is (slow) irreversible deactivation. The main catalytic cycle here is equivalent to that of Figure 5 with n=2. We will work with this example before presenting the results for a general *n*-step cycle.

Using the separability assumption, we present the following simplified version of the main catalytic cycle:

$$Z_{1}\overset{k_{f}}{\underset{k_{r}}{⇄}}Z_{2},$$

with $k_{f}=k_{f,1}+k_{r,2}$ and $k_{r}=k_{r,1}+k_{f,2}$. Applying the QSS principle and the mass conservation law ($Z_{1}+Z_{2}=1$) to the catalyst intermediates of the main cycle gives the following expressions:

(23) $$\begin{array}{cc} Z_{1}= & \frac{k_{r}}{k_{f}+k_{r}}, \end{array}$$

(24) $$\begin{array}{cc} Z_{1}= & 1-Z_{2} \end{array}$$

(25) $$\begin{array}{cc} Z_{1}= & \frac{\frac{1}{k_{f,1}}+\frac{1}{K_{1}k_{f,2}}}{\frac{1}{k_{f,1}}\left(1+\frac{1}{K_{2}}\right)+\frac{1}{k_{f,2}}\left(1+\frac{1}{K_{1}}\right)}, \end{array}$$

where $K_{1}=\frac{k_{f,1}}{k_{r,1}}$ and $K_{2}=\frac{k_{f,2}}{k_{r,2}}$ are apparent equilibrium constants. From this, it can be shown that the main catalytic cycle has (initially, when fresh) QSS rate

(26) $$\begin{array}{cc} R_{\mathrm{qss}}=R_{\mathrm{fresh}}= & \frac{k_{f,1}k_{r}-k_{r,1}k_{f}}{k_{f}+k_{r}}=\frac{k_{r,2}k_{r}-k_{f,2}k_{f}}{k_{f}+k_{r}}, \end{array}$$

(27) $$\begin{array}{cc} = & \frac{1-\frac{1}{K_{1}K_{2}}}{\frac{1}{k_{f,1}}\left(1+\frac{1}{K_{2}}\right)+\frac{1}{k_{f,2}}\left(1+\frac{1}{K_{1}}\right)}. \end{array}$$

In the denominator of this equation, the terms include two separate factors, one kinetic and the other thermodynamic [17]. While the presence of small (deactivation) parameters implies *separability*, the obtained concentrations and QSS rates are only good approximations on a short time scale. The next step is to continue to the next separable block in the three-building-blocks scheme.

## 7. Time-Scale-Based Modeling of Deactivation

As mentioned, catalyst deactivation can be divided into a reversible and an irreversible form. To model deactivation, it is important to distinguish between the two. Irreversible deactivation, for example, is also referred to as aging, hinting at the permanence of this type of deactivation. Reversible deactivation will only affect the activity and production.

We will take a look at the simple example from the previous section. Steps 1 and 2 reflect the main catalytic reaction. Steps 3 and 4 correspond to the reversible and irreversible deactivation processes, respectively. The parameters $k_{f,1}$, $k_{r,1}$, $k_{f,2}$, $k_{r,2}$, $k_{f,d}$, $k_{r,d}$, and $k_{i}$ are apparent kinetic coefficients that, in general, can include concentrations of reactants or products as factors. Typically, $k_{f,1},k_{r,1},k_{f,2},k_{r,2}\gg k_{f,d},k_{r,d}>k_{i}$ so that $O\left(k_{f,d},k_{r,d}\right)=\varepsilon O\left(k_{f,1},k_{r,1},k_{f,2},k_{r,2}\right)$ and $O\left(k_{i}\right)=\delta O\left(k_{f,d},k_{r,d}\right)$.

The variables 0<ε≪1 and 0<δ≤1 determine the order of the time-scale for each of the rates. We show this property by naming the rate equation as follows:

(28) $$\begin{array}{cc} r_{1}= & k_{f,1}Z_{1}-k_{r,1}Z_{2}=\rho_{1}, \end{array}$$

(29) $$\begin{array}{cc} r_{2}= & k_{f,2}Z_{2}-k_{r,2}Z_{1}=\rho_{2}, \end{array}$$

(30) $$\begin{array}{cc} r_{3}= & k_{f,d}Z_{1}-k_{r,d}Z_{0}=\varepsilon \rho_{3}, \end{array}$$

(31) $$\begin{array}{cc} r_{4}= & k_{i}Z_{0}=\varepsilon \delta \rho_{4}. \end{array}$$

The following equations show the relation of the relative concentrations of these intermediates. The first Equation (32) is derived from the law of total mass conservation, and the remaining equations are the rate equations of the other intermediates.

(32) $$\begin{array}{cc} Z_{2} & =1-X-Z_{0}-Z_{1}, \end{array}$$

(33) $$\begin{array}{cc} \frac{dZ_{1}}{dt} & =-\rho_{1}+\rho_{2}-\varepsilon \rho_{3}, \end{array}$$

(34) $$\begin{array}{cc} \frac{dZ_{0}}{dt} & =\varepsilon (\rho_{3}-\delta \rho_{4}), \end{array}$$

(35) $$\begin{array}{cc} \frac{dX}{dt} & =\varepsilon \delta \rho_{4}. \end{array}$$

From the relations above, we see that the relative concentration $Z_{0}$ changes at a time-scale $\tau_{\varepsilon }=\varepsilon t$, which is of an order ε slower than the original time-scale *t*. Take for example $\varepsilon =\frac{1}{3600}$, using timescale *t* over $\tau_{\varepsilon }$ is a question of observing seconds over hours, respectively. Furthermore, the relative concentration $Z_{0}$ changes at a time-scale $\tau_{\delta }=\varepsilon \delta t$, which is of an order εδ slower than the original timescale *t*. This means that although they are present, the deactivation effects are not observable over a timescale *t*.

The introduction of the time scales is the mathematical equivalent of the idea of small parameters, they both imply *separability* of the model. Before starting on the second block, let us see how the information from the first block model is implemented into the model of the whole:

(36) $$\begin{array}{cc} Z_{2} & =1-X-Z_{0}-Z_{1}, \end{array}$$

(37) $$\begin{array}{cc} \frac{dZ_{1}}{dt} & =-\varepsilon \rho_{3}, \end{array}$$

(38) $$\begin{array}{cc} \frac{dZ_{0}}{dt} & =\varepsilon (\rho_{3}-\delta \rho_{4}), \end{array}$$

(39) $$\begin{array}{cc} \frac{dX}{dt} & =\varepsilon \delta \rho_{4}. \end{array}$$

The mass conservation law here is $Z_{1}+Z_{2}=1-X-Z_{0}$, and by implementing this into the model for the main cycle, Equation (24) becomes

(40) $$Z_{1}=\frac{k_{r}}{k_{f}+k_{r}}\left(1-X-Z_{0}\right).$$

This equality, a result of the QSS principle, will replace Equation (37) in the deactivation model. Thus far, we have not needed to calculate the kinetics of the main cycle. Only the QSS values are relevant for our deactivation model. So, while we looked at the simple example from the previous section, all conclusions can be extended to the standard *n*-step form given in Figure 5. This can even be taken a step further, as the main cycle does not even need to be linear. However, the deactivation we discuss in this article is strictly linear, and we will limit the calculations to the form presented in Figure 1.

### 7.1. Modeling Strictly Reversible Deactivation, the Second Block in the Three-Building-Block Scheme

Modeling the second block in the three-building-block scheme, the reversible catalyst deactivation, is equivalent to modeling a scheme without aging. So, here we will discuss the approach to modeling strictly reversible deactivation that is separable from the main catalytic reaction.

In case of solely reversible deactivation, Equations (36)–(40) are adjusted such that δ=0 and X=0:

(41) $$\begin{array}{cc} Z_{1}+Z_{2} & =1-Z_{0}, \end{array}$$

(42) $$\begin{array}{cc} Z_{1} & =\frac{k_{r}}{k_{f}+k_{r}}\left(1-Z_{0}\right), \end{array}$$

(43) $$\begin{array}{cc} \frac{dZ_{0}}{dt} & =\epsilon \rho_{3}. \end{array}$$

Note that once the initial value $Z_{0}\left(0\right)$ is chosen, $Z_{0}\left(0\right)=0$ here, and $Z_{1}\left(0\right)$ and $Z_{2}\left(0\right)$ are fixed. The solution to this model is

(44) $$\begin{array}{cc} Z_{0}\left(t\right)= & \frac{\alpha R_{\mathrm{fresh}}k_{f,d}}{k_{r,d}+\alpha R_{\mathrm{fresh}}k_{f,d}}\left(1-\exp\left(-\left(k_{r,d}+\alpha R_{\mathrm{fresh}}k_{f,d}\right)t\right)\right), \end{array}$$

(45) $$\begin{array}{cc} = & \frac{\alpha K_{d}R_{\mathrm{fresh}}}{1+\alpha K_{d}R_{\mathrm{fresh}}}\left(1-\exp\left(-\left(1+\alpha K_{d}R_{\mathrm{fresh}}\right)k_{r,d}t\right)\right). \end{array}$$

With the two parameters $\alpha K_{d}$ and the experimentally measured $R_{\mathrm{fresh}}$, where

(46) $$\begin{array}{cc} \alpha = & \frac{\frac{1}{k_{f,1}}+\frac{1}{K_{1}k_{f,2}}}{1-\frac{1}{K_{1}K_{2}}}, \end{array}$$

(47) $$\begin{array}{cc} K_{d}= & \frac{k_{f,d}}{k_{r,d}}, \end{array}$$

(48) $$\begin{array}{cc} R_{fresh}= & \frac{1-\frac{1}{K_{1}K_{2}}}{\frac{1}{k_{f,1}}\left(1+\frac{1}{K_{2}}\right)+\frac{1}{k_{f,2}}\left(1+\frac{1}{K_{1}}\right)}, \end{array}$$

the overall rate of the main catalytic cycle follows:

(49) $$\begin{array}{cc} R(t)= & R_{\mathrm{fresh}}\left(1-Z_{0}\left(t\right)\right), \end{array}$$

(50) $$\begin{array}{cc} = & R_{\mathrm{fresh}}\underset{\mathrm{deactivation}\mathrm{factor}}{\underbrace{\frac{1+\alpha K_{d}R_{\mathrm{fresh}}\exp\left(-\left(1+\alpha K_{d}R_{\mathrm{fresh}}\right)k_{r,d}t\right)}{1+\alpha K_{d}R_{\mathrm{fresh}}}}}. \end{array}$$

The physical meaning of α is the following, i.e., the apparent time of relaxation that corresponds to the main catalytic cycle.

If the catalytic cycle is irreversible, α simplifies to $1/k_{f,1}$ and $R_{\mathrm{fresh}}$ to $1/(1/k_{f,1}+1/k_{f,2})$. In this case, for the general *n*-step cycle, we refer to Appendix A. Implementing just the two separable blocks, the rate is given by multiplication of the rate from the first (main cycle) block and a deactivation factor.

In the definitions, $R_{\mathrm{fresh}}$ does not depend on the deactivation parameters. At the same time, the deactivation factor depends on the apparent deactivation parameters $K_{d}$ and $k_{r,d}$, and on the values α and $R_{\mathrm{fresh}}$ of the main cycle. Therefore, the deactivation factor encapsulates the apparent parameters of the main cycle. This is the simplest case of hierarchical separation, in which the second factor depends on the apparent parameters of the first.

### 7.2. Combination of Reversible and Irreversible Deactivation

While the strictly reversible deactivation model was easy to derive, the combination of reversible and irreversible deactivation needs to be guided carefully. Let us look at the following cases δ=1 and δ≪1.

In the first case δ=1, the irreversible and reversible deactivation have the same time scale. In a graphical representation, this would mean the graph can only be split into two segments: the main catalytic cycle and deactivation. This means that aging is not *separable* from the reversible deactivation. Our mathematical model would look as follows:

(51) $$\begin{array}{cc} Z_{1}+Z_{2} & =1-X-Z_{0}, \end{array}$$

(52) $$\begin{array}{cc} Z_{1} & =\frac{k_{r}}{k_{f}+k_{r}}\left(1-X-Z_{0}\right), \end{array}$$

$$\begin{array}{cc} \frac{dZ_{0}}{dt} & =\varepsilon (\rho_{3}-\rho_{4}), \end{array}$$

(53) $$\begin{array}{cc} \; & =k_{f,d}Z_{1}-k_{r,d}Z_{0}-k_{i}Z_{0}, \end{array}$$

$$\begin{array}{cc} \frac{dX}{dt} & =\varepsilon \rho_{4}, \end{array}$$

(54) $$\begin{array}{cc} \; & =k_{i}Z_{0}. \end{array}$$

The solution here is

(55) $$\begin{array}{cc} Z_{0}\left(t\right)= & C_{1}e^{-\lambda_{1}t}-C_{1}e^{-\lambda_{2}t}, \end{array}$$

(56) $$\begin{array}{cc} X(t)= & C_{2}e^{-\lambda_{1}t}-\left(C_{2}+1\right)e^{-\lambda_{2}t}+1, \end{array}$$

where

(57) $$\begin{array}{cc} \lambda_{1}= & \frac{\left(1+\alpha R_{\mathrm{fresh}}K_{d}\right)k_{r,d}+k_{i}}{2}-\frac{\sqrt{{\left(\left(1+\alpha R_{\mathrm{fresh}}K_{d}\right)k_{r,d}+k_{i}\right)}^{2}-4\alpha K_{d}R_{\mathrm{fresh}}k_{r,d}k_{i}}}{2}, \end{array}$$

(58) $$\begin{array}{cc} \lambda_{2}= & \frac{\left(1+\alpha R_{\mathrm{fresh}}K_{d}\right)k_{r,d}+k_{i}}{2}+\frac{\sqrt{{\left(\left(1+\alpha R_{\mathrm{fresh}}K_{d}\right)k_{r,d}+k_{i}\right)}^{2}-4\alpha K_{d}R_{\mathrm{fresh}}k_{r,d}k_{i}}}{2}, \end{array}$$

(59) $$\begin{array}{cc} C_{1}= & \frac{\alpha R_{\mathrm{fresh}}k_{f,d}}{\sqrt{{\left(\left(1+\alpha R_{\mathrm{fresh}}K_{d}\right)k_{r,d}+k_{i}\right)}^{2}-4\alpha K_{d}R_{\mathrm{fresh}}k_{r,d}k_{i}}}, \end{array}$$

(60) $$\begin{array}{cc} C_{2}= & -\frac{1}{2}-\frac{\left(1+\alpha R_{\mathrm{fresh}}K_{d}\right)k_{r,d}+k_{i}}{2\sqrt{{\left(\left(1+\alpha R_{\mathrm{fresh}}K_{d}\right)k_{r,d}+k_{i}\right)}^{2}-4\alpha K_{d}R_{\mathrm{fresh}}k_{r,d}k_{i}}}. \end{array}$$

The the overall rate for the main catalytic reaction is

(61) $$\begin{array}{cc} R(t)= & R_{\mathrm{fresh}}\left(1-X\left(t\right)-Z_{0}\left(t\right)\right), \end{array}$$

(62) $$\begin{array}{cc} = & R_{\mathrm{fresh}}\left(-\left(C_{1}+C_{2}\right)e^{-\lambda_{1}t}+\left(1+C_{1}+C_{2}\right)e^{-\lambda_{2}t}\right). \end{array}$$

Since δ=1, the reversible and irreversible deactivation are not separable and, thus, the model is based on a two-block scheme, where block one is the main catalytic cycle and block two is the general catalyst deactivation. Given that there are two blocks, we again see a multiplications of two factors, the rate for block one and factor linked to deactivation.

The second case δ≪1 introduces an even smaller parameter or a third time scale. As such, here, we can introduce a third block to describe the aging.

One may assume a QSS situation for the reversible deactivation if the frame of study lies far in time. A QSS assumption for the reversible deactivation implies that we may substitute Equations (37) and (38) with appropriate algebraic expressions. So, $Z_{0}$ will be constant in time and equal to the limit value of Equation (45), scaled to uphold the law of mass conservation ($Z_{0}+Z_{1}+Z_{2}=1-X$),

(63) $$\begin{array}{cc} Z_{2} & =1-X-Z_{0}-Z_{1}, \end{array}$$

(64) $$\begin{array}{cc} Z_{1} & =\frac{k_{r}}{k_{f}+k_{r}}\left(1-X-Z_{0}\right), \end{array}$$

(65) $$\begin{array}{cc} Z_{0} & =\frac{\alpha K_{d}R_{\mathrm{fresh}}}{1+\alpha K_{d}R_{\mathrm{fresh}}}\left(1-X\right), \end{array}$$

$$\begin{array}{cc} \frac{dX}{dt} & =\varepsilon \rho_{4}, \end{array}$$

(66) $$\begin{array}{cc} \; & =k_{i}Z_{0}. \end{array}$$

The solution to this system of equations is

(67) $$X\left(t\right)=1-\exp\left(-\frac{\alpha K_{d}R_{fresh}}{\alpha K_{d}R_{fresh}+1}k_{i}t\right).$$

To determine the rate of this three-block scheme, we take

(68) $$\begin{array}{cc} R(t)= & R_{\mathrm{fresh}}\left(1-X\left(t\right)-Z_{0}\left(t\right)\right), \end{array}$$

(69) $$\begin{array}{cc} = & R_{\mathrm{fresh}}\frac{1}{1+\alpha K_{d}R_{\mathrm{fresh}}}\exp\left(-\frac{\alpha K_{d}R_{\mathrm{fresh}}}{\alpha K_{d}R_{\mathrm{fresh}}+1}k_{i}t\right), \end{array}$$

in which three parameters occur: $R_{\mathrm{fresh}}$, $\alpha K_{d}$, and $k_{i}$.

This rate equation is a multiplication of the QSS rate of the first block, the QSS rate of the second block, and a factor for the irreversible deactivation. Note that it was mentioned that this approach is ideal for a long-term study.

To get a better approximation of the overall rate, we may assume that the rate for the three-block scheme is a multiplication of the rate of the two-block scheme with the factor for irreversible deactivation. This results in the following equation:

(70) $$\;\;\;\;R\left(t\right)=R_{\mathrm{fresh}}\underset{\mathrm{reversible}\mathrm{deactivation}\mathrm{factor}}{\underbrace{\frac{1+\alpha K_{d}R_{\mathrm{fresh}}\exp\left(-\left(1+\alpha K_{d}R_{\mathrm{fresh}}\right)k_{r,d}t\right)}{1+\alpha K_{d}R_{\mathrm{fresh}}}}}\underset{\mathrm{irreversible}\mathrm{deactivation}\mathrm{factor}}{\underbrace{\exp\left(-\frac{\alpha K_{d}R_{\mathrm{fresh}}}{1+\alpha K_{d}R_{\mathrm{fresh}}}k_{i}t\right)}}.$$

The irreversible deactivation factor is a function of all apparent parameters, of the main cycle α and $R_{\mathrm{fresh}}$, reversible deactivation $K_{d}$, and irreversible deactivation $k_{i}$, but not of $k_{r,d}$. This case is another example of hierarchical separation, in which each level occurs as a function of all previous ones.

For the general *n*-step cycle, we refer to Appendix A. Due to linearity, we can calculate the exact solution for Equation (70). The exact solution will be a sum of three terms, all of which depend on all apparent parameters, while our approximation is a product of three factors only dependent on the apparent parameters of the respective and previous blocks.

## 8. Application

In this section, we are going to apply our approach to determine curves that will describe different sets of experimental data from catalyst deactivation. Generally, the whole catalytic process is complex and includes three subprocesses, i.e., catalyst activation, the catalytic cycle and catalyst deactivation, reversible and irreversible. Regarding catalyst activation, during which the catalytic center is formed, it is typically the fast adsorption–catalytic process. The catalyst activation process is overly sensitive to conditions of the catalyst preparation, and reliable information is sparse or completely absent. Thus, our analysis is only about the main catalytic cycle accompanied by catalyst deactivation. We selected examples for illustrating our approach based on the following criteria:

1. Reliable information;
2. Data with a clearly distinguished reversible deactivation process;
3. Different types of kinetic reactors should be represented, i.e., CSTR and PFR.

### 8.1. Catalyst Deactivation in a Differential Reactor

**Figure 6:**
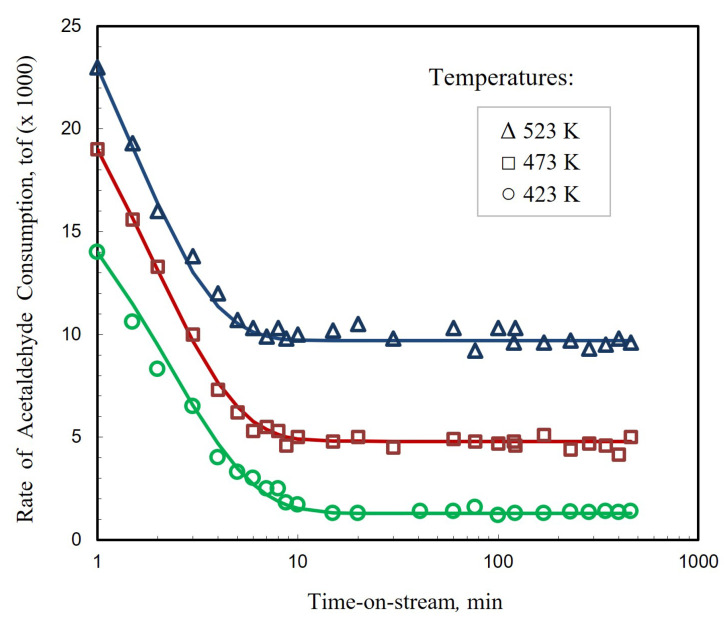
Time-on-stream dependence of the acetaldehyde rate change on $TiO_{2}$(tof, $s^{-1}$). The bullet points are data reconstructed from [18] and the lines are R(t) as calculated by Equation (79) (or (75)).

First, we analyzed the differential reactor data on rapid catalyst deactivation observed for aldol condensation with dehydration of acetaldehyde to produce crotonaldehyde on $TiO_{2}$ anatase [18]. Secondary condensations that deposit nonvolatile organic species on the catalyst surface are responsible for the initial rapid catalyst deactivation. After 15–20 min, the process is stabilized at an apparent steady-state level (Figure 6). Therefore, in this case, the deactivation process is reversible, and no further irreversible deactivation is observed. Then, it was found [18] that the deactivation rate is independent of the acetaldehyde concentration.

Since the detailed mechanism of deactivation is not presented in [18], the equation of reversible catalyst deactivation can be assumed to be similar to Equation (9); it reflects the transformation of the active surface coverage $Z_{A}$ to the surface “nonvolatile deposits”, $Z_{D}$,

(71) $$\frac{dZ_{A}}{dt}=-k_{d}Z_{A}+k_{r}Z_{D},$$

where $k_{d}$ and $k_{r}$ are apparent parameters of catalyst deactivation and self-regeneration, respectively.

According to Equation (11), $Z_{A}=Z_{A}^{0}\left(1-Z_{D}\right)$, and, since $Z_{A}^{0}=1$,

(72) $$\frac{d(1-Z_{D})}{dt}=-k_{d}\left(1-Z_{D}\right)+k_{r}Z_{D}.$$

As given in Equations (13) and (49),

(73) $$a\left(t\right)=\frac{R(t)}{R_{\mathrm{fresh}}}=1-Z_{D}\left(t\right).$$

Substituting this into Equation (72) results in the following equation for the relative activity,

(74) $$\frac{da(t)}{dt}=-k_{d}a\left(t\right)+k_{r}\left(1-a\left(t\right)\right).$$

The analytic solution of this equation is

(75) $$a\left(t\right)=\frac{R(t)}{R_{\mathrm{fresh}}}=\frac{1}{k_{d}+k_{r}}\left(k_{r}+k_{d}\exp\left(-\left(k_{d}+k_{r}\right)\left(t-t_{0}\right)\right)\right),$$

where $R_{\mathrm{fresh}}=R\left(t_{0}\right)$, and it was stated in [18] that $t_{0}=1\min$, which can also be observed in Figure 6.

Kinetic parameters of deactivation are presented in the following Table 1.

The third parameter in Table 1 is the steady-state level of the reaction rate. It is an asymptotic solution of Equation (75) at t→∞, and

(76) $$a_{s}=\frac{R_{s}}{R_{\mathrm{fresh}}}=\frac{k_{r}}{k_{d}+k_{r}}.$$

Parameter $a_{s}$ can be also found from Equation (74) at $\frac{da}{dt}=0$. Then, Equation (74) can be rewritten similarly to Equation (18), so that parameter $k_{r}$ is expressed in terms of $a_{s}$ using Equation (76),

(77) $$\frac{da(t)}{dt}=-k_{d}\frac{a\left(t\right)-a_{s}}{1-a_{s}}.$$

The solution of Equation (77) is as follows:

(78) $$a\left(t\right)=a_{s}+\left(1-a_{s}\right)\exp\left(-\frac{k_{d}}{1-a_{s}}\left(t-t_{0}\right)\right).$$

Due to $a=\frac{R(t)}{R_{\mathrm{fresh}}}$ and $a_{s}=\frac{R_{s}}{R_{\mathrm{fresh}}}$, the solution can also be written as

(79) $$R\left(t\right)=R_{s}+\left(R_{\mathrm{fresh}}-R_{s}\right)\exp\left(-\frac{k_{d}}{1-R_{s}/R_{\mathrm{fresh}}}\left(t-t_{0}\right)\right).$$

Both this equation and Equation (75) can be used to describe the experimental data. As such, we present the analytic curve along with the data points in Figure 6. An excellent fitting is observed.

### 8.2. Catalyst Deactivation in an Integral Reactor

The integral reactor data of catalyst deactivation for crotonaldehyde hydrogenation on supported metal catalysts was analyzed [16,19,20]. In this case, the catalyst deactivation process is reversible due to self-regeneration by hydrogen, which is in excess within the reaction mixture.

The problem with describing the integral reactor data on deactivation is that the catalyst activity, as well as the reagent concentrations, changes along the catalyst bed, but only concentrations at the reactor exit are measured. Therefore, our model for catalyst deactivation must be modified.

**Figure 7:**
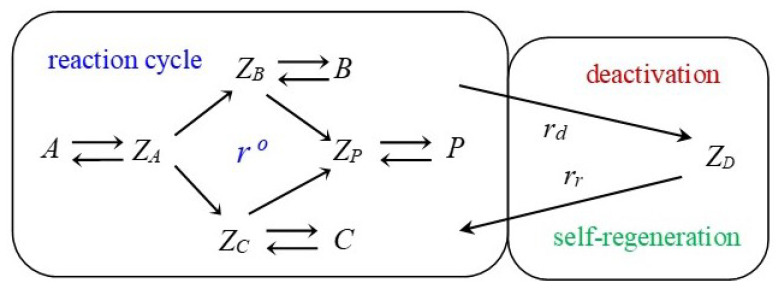
Schematic view of the crotonaldehyde hydrogenation. A is crotonaldehyde, ${\mathrm{CH}}_{3}–\mathrm{CH}=\mathrm{CH}–\mathrm{CH}=O$; B is butyraldehyde, ${\mathrm{CH}}_{3}–{\mathrm{CH}}_{2}–{\mathrm{CH}}_{2}–\mathrm{CH}=O$; C is crotyl alcohol, ${\mathrm{CH}}_{3}–\mathrm{CH}=\mathrm{CH}–{\mathrm{CH}}_{2}–\mathrm{OH}$; P is butanol, ${\mathrm{CH}}_{3}–{\mathrm{CH}}_{2}–{\mathrm{CH}}_{2}–{\mathrm{CH}}_{2}–\mathrm{OH}$.

In this section, the mechanism of crotonaldehyde hydrogenation is modified by adding catalyst deactivation and self-regeneration steps (Figure 7).

In [16], based on the previous study [19], adsorbed crotonaldehyde $Z_{A}$ is assumed to be the prevailing surface intermediate, so that all surface sites are completely covered by crotonaldehyde ($Z_{A}$ ∼ 1). Therefore, the surface coverage of other species can be neglected ($Z_{E}$ ∼ 0). This greatly facilitates the analysis of the experimental data and the derivation of the catalyst deactivation equations as well.

Reactant concentrations are changed along the catalyst bed length (ξ) and expressed as follows:

(80) $$\begin{array}{cc} C_{A}\left(\xi \right)= & C_{A0}\left(1-x\left(\xi \right)\right), \end{array}$$

(81) $$\begin{array}{cc} C_{E}\left(\xi \right)= & C_{A0}x\left(\xi \right), \end{array}$$

where $x=\frac{\left(C_{A0}-C_{A}\right)}{C_{A0}}$ is the crotonaldehyde conversion x(ξ,t); $C_{A}$ and $C_{A0}$ are crotonaldehyde concentration and inlet concentration, respectively; $C_{E}=C_{B}+C_{C}+C_{P}$; and ξ is a dimensionless length coordinate.

Since $dC_{A}=-C_{A0}dx$, the corresponding differential equation has the form

(82) $$\begin{array}{cc} \frac{dC_{A}}{d\xi }= & -k_{s}\tau C_{H}Z_{A}, \end{array}$$

(83) $$\begin{array}{cc} \frac{dx}{d\xi }= & k_{s}\tau \frac{C_{H}}{C_{A0}}Z_{A}, \end{array}$$

where $k_{s}$ is the apparent reaction rate constant and τ is the contact time.

According to the scheme in Figure 7, the equation of catalyst deactivation represents the dynamics of $Z_{A}$ as

(84) $$\frac{dZ_{A}}{dt}=-k_{d}Z_{A}C_{E}+k_{r}Z_{D}C_{H}.$$

Deactivation in crotyl alcohol formation can be ascribed to the generation of strongly chemisorbed asymmetric carboxylate species [20], which usually exist as dimers.

According to Equation (11), $Z_{A}=Z_{A}^{0}\left(1-Z_{D}\right)$. Adsorption steps (see Figure 7) are fast and reversible; thus, it is reasonable to consider them under equilibrium conditions.

Therefore, $Z_{A}^{0}=K_{A}C_{A}=K_{A}C_{A0}\left(1-x\right)$; then, Equation (83) becomes

(85) $$\begin{array}{cc} \frac{dx}{d\xi }= & k_{S}\tau \frac{C_{H}}{C_{A0}}Z_{A},\\ = & k_{S}\tau \frac{C_{H}}{C_{A0}}K_{A}C_{A0}\left(1-x\right)\left(1-Z_{D}\right),\\ = & k_{eff}\tau \left(1-x\right)\left(1-Z_{D}\right). \end{array}$$

A similar transformation of Equation (84), given $Z_{A}=Z_{A}^{0}\left(1-Z_{D}\right)$ and $C_{E}=C_{A0}x$ from Equation (81), leads to

(86) $$Z_{A}^{0}\frac{d(1-Z_{D})}{dt}=-k_{d}Z_{A}^{0}\left(1-Z_{D}\right)C_{A0}x+k_{r}Z_{D}C_{H}.$$

The right-hand side of (86) represents the reaction rate, so similarly to Equation (49), $R\left(t\right)=R_{\mathrm{fresh}}\left(1-Z_{D}\right)$, and again, the relative activity *a* is proportional to $Z_{D}$, as in Equation (73),

(87) $$a\left(t\right)=\frac{R(t)}{R_{\mathrm{fresh}}}=1-Z_{D}\left(t\right).$$

Due to the excess of hydrogen [19], $C_{A0}\approx 1\%$, $C_{H}\approx 99\%$, and $Z_{A}^{0}∼1$. These features give the possibility to simplify Equations (85) and (86) and express them in terms of the relative activity *a*,

(88) $$\begin{array}{cc} \frac{dx}{d\xi }= & k_{eff}\tau \left(1-x\right)a, \end{array}$$

(89) $$\begin{array}{cc} \frac{da}{dt}= & -k_{d}C_{A0}xa+k_{r}C_{H}\left(1-a\right). \end{array}$$

**Figure 8:**
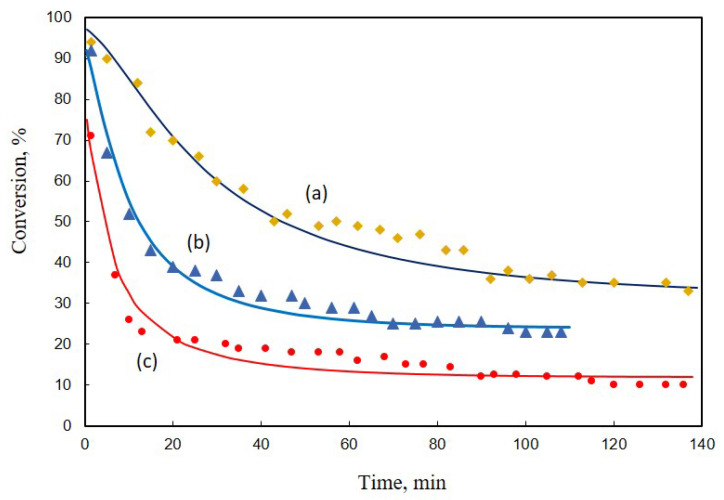
Application of the model to the catalyst deactivation in the reaction of crotonaldehyde hydrogenation, after reduction at 443 K, during 4 h. The bullet points are data reconstructed from [16] and the lines are solutions of Equation (92). (a) Catalyst precursor $\mathrm{Pt}{\left({\mathrm{NH}}_{3}\right)}_{4}{\left({\mathrm{NO}}_{3}\right)}_{2}$; testing after second reduction. (b) Catalyst precursor $H_{2}\mathrm{Pt}{\mathrm{Cl}}_{6}$; testing after first reduction. (c) Catalyst precursor $\mathrm{Pt}{\left({\mathrm{NH}}_{3}\right)}_{4}{\left({\mathrm{NO}}_{3}\right)}_{2}$; testing after first reduction.

In [16], the dynamics of catalyst activity was presented as a time-dependence of crotonaldehyde conversion at the exit of the catalyst bed X(t)=x(ξ=1,t), Figure 8.

Thus, X(t) can be determined by solving Equation (88),

(90) $$\begin{array}{cc} X= & 1-\exp\left(-k_{eff}\tau \langle a\rangle \right) \end{array}$$

(91) $$\begin{array}{cc} \langle a\rangle = & -\frac{\ln(1-X)}{k_{eff}\tau }, \end{array}$$

where 〈a〉 is the integral activity of the catalyst bed. It can be found by integration of Equation (89).

The method of converting the system of Equations (88) and (89) into a single equation for the exit conversion, *X*, is described in detail in [13],

(92) $$\frac{dX}{dt}=k_{d}C_{A0}\left(1-X\right)\left(X+\ln\left(1-X\right)\right)\left(1-\frac{\ln(1-X_{s})}{\ln(1-X)}\right).$$

Here, $X_{s}$ is the steady-state conversion at which rates of deactivation and self-regeneration are equal.

There is no analytical solution for Equation (92); therefore, we solve it by numerical integration, comparing the results with the experimental data (Figure 8). Model parameters (at $C_{A0}=0.01\mathrm{mol}/\mathrm{mol})$ are presented in Table 2.

## 9. Discussion and Conclusions

In this article, we reviewed the history of the derivation of the kinetic model of catalyst deactivation, focusing specifically on the modified phenomenological models. Based on a detailed analysis of the application of the QSS assumption, we justified the *separability* principle, which is considered in the description of catalyst deactivation.

We assumed a three-block model of catalyst deactivation. The three blocks are the main catalytic cycle, the one-step reversible deactivation, and the one-direction irreversible deactivation. Assuming the QSS of the main cycle, we then set up a 2-step cycle example. The separability of the model is replicated in the separable factors that we find in the rate equation. For a three-building-block scheme, the result is

(93) $$R\left(t\right)=R_{\mathrm{fresh}}\psi_{d}\psi_{a},$$

where $\psi_{d}$ is the reversible deactivation factor and $\psi_{a}$ is the irreversible (aging) deactivation factor.

The reversible deactivation factor $\psi_{d}$ is a function of the apparent parameters of the main cycle α and $R_{\mathrm{fresh}}$, and of the apparent parameters of the reversible deactivation $K_{d}$ and $k_{r,d}$. The irreversible deactivation factor $\psi_{a}$ is a function of all apparent parameters of the main cycle α and $R_{\mathrm{fresh}}$, reversible deactivation $K_{d}$, and irreversible deactivation $k_{i}$, but not of $k_{r,d}$. These dependencies of the factors exhibit hierarchical separation according to which each factor depends only on the apparent parameters of the previous ones and of itself.

This equation is obtained under the assumption that the main cycle is a single-route catalytic reaction with a linear mechanism. However, heuristically, this equation can be tested beyond this assumption. In the next papers, we shall apply this three-factor equation for the detailed theoretical analysis and description of experimental data.

The obtained equation is applied successfully to describe the literature data on the reversible catalyst deactivation process in dehydration of acetaldehyde over $TiO_{2}$ anatase and in crotonaldehyde hydrogenation on supported metal catalysts. In the future, we will apply our approach towards describing systems that we consider potential candidates. We mention these systems as additional references [21,22].

## Figures and Tables

**Table 1:** Kinetic parameters of deactivation per given temperature.

T, K	523	473	423
$k_{d}$, $\min^{-1}$	0.4	0.4	0.4
$k_{r}$, $\min^{-1}$	0.292	0.135	0.041
$R_{s}\;\cdot \;10^{3}$, $s^{-1}$	9.7	4.8	1.3

**Table 2:** Model parameters of deactivation per catalyst precursor.

Catalyst	(a)	(b)	(c)
$k_{d}$, $\min^{-1}$	12	34	60
$X_{s}$, %	32	24	12

## Data Availability

The data points in Figure 6 and Figure 8 were reconstructed from the data in articles [18] and [16], respectively. The curves in Figure 6 and Figure 8 were calculated using our model and the data from articles [18] and [16], respectively.

## Abbreviations

The following abbreviations are used in this manuscript:

QSS	quasi-steady-state

## Appendix A. General *N*-Step Cycle

Writing [a] for the value in {1,⋯,n} that is equal to *a* modulo *n*, see [17], the generalization of (27) is

(A1) $$\begin{array}{cc} \frac{1-\frac{1}{K_{1}\cdots K_{n}}}{R_{\mathrm{fresh}}}= & \sum_{i=1}^{n}\frac{1}{k_{f,i}}\left(1+\frac{1}{K_{\left[i-1\right]}}+\frac{1}{K_{\left[i-1\right]}K_{\left[i-2\right]}}+\cdots +\frac{1}{K_{\left[i-1\right]}\cdots K_{\left[i-(n-1)\right]}}\right)\\ = & \sum_{i,j=1}^{n}\frac{T_{ij}}{k_{f,i}}, \end{array}$$

so that

(A2) $$R_{\mathrm{fresh}}=\frac{1-\frac{1}{K_{1}\cdots K_{n}}}{\sum_{i,j=1}^{n}\frac{T_{ij}}{k_{f,i}}},\;T_{ij}=\frac{1}{\prod_{k=1}^{j-1}K_{\left[i-k\right]}}.$$

Generalizing (25), the concentrations are then determined by

(A3) $$\frac{1-\frac{1}{K_{1}\cdots K_{n}}}{R_{\mathrm{fresh}}}Z_{j}=\sum_{i=1}^{n}\frac{T_{i,[i-j+1]}}{k_{f,i}}.$$

To take the deactivation into account, we need the parameter

(A4) $$\theta_{1}=\frac{\sum_{i=1}^{n}\frac{T_{i,i}}{k_{f,i}}}{\sum_{i,j=1}^{n}\frac{T_{ij}}{k_{f,i}}},$$

which is the relative coverage of $Z_{1}$ compared to the sum of all $Z_{i}$ present in the main *n*-step reaction, namely, for those labeled i=1,⋯,n. Expression (50) generalizes to

(A5) $$R\left(t\right)=\frac{1-\frac{1}{K_{1}\cdots K_{n}}}{\sum_{i,j=1}^{n}\frac{T_{ij}}{k_{f,i}}}\frac{1+\theta_{1}K_{d}\exp\left(-\left(1+\theta_{1}K_{d}\right)k_{r,d}t\right)}{1+\theta_{1}K_{d}}.$$

For the general *n*-step cyclic mechanism, (70) is

(A6) $$R\left(t\right)=\frac{1-\frac{1}{K_{1}\cdots K_{n}}}{\sum_{i,j=1}^{n}\frac{T_{ij}}{k_{f,i}}}\frac{1+\theta_{1}K_{d}\exp\left(-\left(1+\theta_{1}K_{d}\right)k_{r,d}t\right)}{1+\theta_{1}K_{d}}\exp\left(-\frac{\theta_{1}K_{d}}{1+\theta_{1}K_{d}}k_{i}t\right).$$

## Appendix B. λ Analysis

The solution for a 2-step cyclic mechanism as presented in Section 5,

(A7) $$R=C_{1}e^{-\lambda_{1}t}+C_{2}e^{-\lambda_{2}t}+C_{3}e^{-\lambda_{3}t},$$

where

(A8) $$\begin{array}{cc} C_{1}= & \left(1-\alpha R_{\mathrm{fresh}}\right)\left(k_{f}+k_{r}+\alpha K_{d}R_{\mathrm{fresh}}k_{r,d}\right)+\ldots \end{array}$$

(A9) $$\begin{array}{cc} \lambda_{1}= & -\left(k_{f}+k_{r}\right)-\left(1-\alpha R_{\mathrm{fresh}}\right)K_{d}k_{r,d}+\ldots \end{array}$$

(A10) $$\begin{array}{cc} C_{2}= & -\left(1-\alpha R_{\mathrm{fresh}}\right)\alpha K_{d}R_{\mathrm{fresh}}k_{r,d}+\ldots \end{array}$$

(A11) $$\begin{array}{cc} \lambda_{2}= & -\left(1+\alpha K_{d}R_{\mathrm{fresh}}\right)k_{r,d}+\ldots \end{array}$$

(A12) $$\begin{array}{cc} C_{3}= & -\frac{\left(1-\alpha R_{\mathrm{fresh}}\right)\alpha K_{d}R_{f}}{{\left(1+\alpha K_{d}R_{\mathrm{fresh}}\right)}^{2}}k_{i}+\ldots \end{array}$$

(A13) $$\begin{array}{cc} \lambda_{3}= & -\frac{\alpha K_{d}R_{\mathrm{fresh}}}{1+\alpha K_{d}R_{\mathrm{fresh}}}k_{i}+\ldots \end{array}$$

The first eigenvalue represents the fast-reaching of quasi-steady-state by the catalytic main cycle; the second is determined by the reversible deactivation; the third is determined by the irreversible deactivation.
